# Neuronal ICAM-5 Inhibits Microglia Adhesion and Phagocytosis and Promotes an Anti-inflammatory Response in LPS Stimulated Microglia

**DOI:** 10.3389/fnmol.2017.00431

**Published:** 2017-12-22

**Authors:** Sonja Paetau, Taisia Rolova, Lin Ning, Carl G. Gahmberg

**Affiliations:** Laboratory of CG Gahmberg, Division of Biochemistry and Biotechnology, Department of Biosciences, University of Helsinki, Helsinki, Finland

**Keywords:** ICAM-5, microglia, adhesion, phagocytosis, TNF-α, ICAM-1, integrin

## Abstract

The intercellular adhesion molecule-5 (ICAM-5) regulates neurite outgrowth and synaptic maturation. ICAM-5 overexpression in the hippocampal neurons induces filopodia formation *in vitro*. Since microglia are known to prune supernumerous synapses during development, we characterized the regulatory effect of ICAM-5 on microglia. ICAM-5 was released as a soluble protein from *N*-methyl-D-aspartic acid (NMDA)-treated neurons and bound by microglia. ICAM-5 promoted down-regulation of adhesion and phagocytosis *in vitro*. Microglia formed large cell clusters on ICAM-5-coated surfaces whereas they adhered and spread on the related molecule ICAM-1. ICAM-5 further reduced the secretion of the proinflammatory cytokines tumor necrosis factor α (TNF-α) and interleukin 1β (IL-1β), but on the contrary induced the secretion of the anti-inflammatory IL-10 from lipopolysaccharide (LPS) stimulated microglia. Thus, ICAM-5 might be involved in the regulation of microglia in both health and disease, playing an important neuroprotective role when the brain is under immune challenges and as a “don’t-eat-me” signal when it is solubilized from active synapses.

## Introduction

The intercellular adhesion molecule-5 (ICAM-5), also known as telencephalin, is a membrane-spanning adhesion molecule that regulates neuronal development is several ways (Tian et al., 2000b, 2007; Matsuno et al., 2006; Gahmberg et al., 2014). ICAM-5 is expressed on the dendrites and soma of neurons of the telencephalon, with the highest level of expression in the glutamatergic neurons of the hippocampus and cerebral cortex. The extracellular part contains nine immunoglobulin domains that bind several ligands (Yoshihara et al., 1994; Tian et al., 1997; Zhang et al., 2008; Furutani et al., 2012; Recacha et al., 2014). During the initial contact between the pre- and the postsynaptic terminals, ICAM-5 binds β1 integrins on the presynaptic element and stabilizes the contact sites (Ning et al., 2013). As the spines mature, ICAM-5 migrates away from the postsynaptic density to the spine shafts. During early development, ICAM-5 induces neurite sprouting, supposedly through homophilic binding (Tian et al., 2000b). On mature dendrites, ICAM-5 promotes filopodia formation, but subsequently retards spine maturation (Matsuno et al., 2006). ICAM-5 inhibits the maturation process, but under regulated conditions maturation must be granted. In the glutamatergic synapses where ICAM-5 is expressed, *N*-methyl-D-aspartic acid (NMDA) receptor signaling removes the ICAM-5 block. This leads to shedding of the extracellular part of ICAM-5 (Tian et al., 2007). ICAM-5 is cleaved by matrix metalloproteinases (MMP), mainly MMP-2 and -9, generating two major soluble fragments around 100 kDa (Tian et al., 2007; Conant et al., 2010). Once the ectodomain is cleaved, the cytoplasmic tail is released from the actin-linking protein α-actinin. The interaction with α-actinin is important for neurite sprouting and elongation and spine maturation (Nyman-Huttunen et al., 2006; Ning et al., 2015).

Microglia express integrins that have been shown to bind ICAM-5 in other cells (Akiyama and McGeer, 1990). The α5β1 integrin [very late antigen-5, (VLA-5)] on neurons and the αLβ2 [leukocyte function associated antigen-1 (LFA-1)] on leukocytes have been demonstrated to act as receptors for ICAM-5 (Tian et al., 1997; Ning et al., 2013). Our laboratory has previously shown that ICAM-5 has an immunosuppressive effect on T cells (Tian et al., 2008).

Microglia are macrophage-like cells in the brain that respond to brain injury by secreting proinflammatory cytokines. Inflammation is associated with most traumatic, neurodegenerative and neurodevelopmental disorders, where microglia are key players. At the resolving phase of the injury, microglia adopt a neuroprotective phenotype and the cytokine profile shifts toward anti-inflammatory. Cytokines considered as proinflammatory are for example IL-1, IL-6, TNF-α, and interferon-γ. Classical components of the anti-inflammatory cytokine profile are IL-4, IL-10 and transforming growth factor-β (Smith et al., 2012). Although microglia are the resident immune cells of the brain, they have been shown to contribute to much more than immune functions in the central nervous system. They can regulate synaptic plasticity, prune synaptic structures, provide neuronal guidance and secrete neurotrophic factors (Paolicelli et al., 2011; Hong et al., 2016). Microglia can make direct contact with both pre- and postsynaptic elements and modulate the collapse or maturation of synapses (Wake et al., 2009; Miyamoto et al., 2016). These phagocytic cells remove excess synapses through a process that is regulated by components of the complement cascade (Stevens et al., 2007; Schafer et al., 2012). If ICAM-5 is bound to microglia, it could potentially regulate several aspects of the above-mentioned microglia functions. Both ICAM-5 and microglia play a role in immunological and neuroplastic events. Therefore, we have now studied the effect of ICAM-5 on microglia and gained novel insights into the regulatory effect that neurons have on microglia.

## Materials and Methods

### Reagents and Antibodies

The polyclonal rabbit antibody 1000J [1:1000 in western blot (WB) or immunofluorescence (IF)] and monoclonal mouse 246H (1:2000 in WB) and 179D [1 μg in immunoprecipitation (IP)] against immunoglobulin (Ig)-like domains 1–2 (D1-2) of ICAM-5 were kind gifts from P. Kilgannon (ICOS corporation, Seattle, WA, United States) (Tian et al., 2000a; Ning et al., 2013). Integrin β1 monoclonal antibody TS2/16 was a kind gift from Dr. T. A. Springer (Harvard Medical School, Boston, MA, United States), 1:500 in WB (Ning et al., 2013).

The following antibodies were purchased: integrin β2 (10E12, ab86457, Abcam, Cambridge, United Kingdom, 1:200 in IF and 1:1000 in WB), integrin β1 (ab52971, Abcam, 1:150 in IF, 1 μg in IP), Iba1 (Wako, Japan, 1:100 in IF), β-actin (5060, Sigma–Aldrich, St. Louis, MO, United States, 1:2000 in WB), mouse IgG (Mi4 ms IgG1, Merck Millipore, Burlington, MA, United States, 1 μg in IP), ICAM-1 function blocking antibody (LEAF^TM^ purified anti-mouse CD54, YN1/1.7.4, BioLegend, San Diego, CA, United States) and the isotype control (LEAF^TM^ purified rat IgG2bκ, RTK4530, BioLegend). Horseradish peroxidase (HRP)-conjugated secondary antibodies (Cell Signaling Technology, Danvers, MA, United States, 1:3000) were used for WB. For the IF, we used Alexa Fluor 488-, Alexa Fluor 633- (Molecular Probes, Thermo Fisher Scientific, Waltham, MA, United States, 1:300) and cy3-conjugated (Jackson ImmunoResearch, West Grove, PA, United States, 1:300) secondary antibodies, Alexa Fluor 633-conjugated phalloidin (1:100), and 4′,6-Diamidine-2′-phenylindole dihydrochloride (DAPI, 1:10 000, Molecular Probes).

**FIGURE 1:**
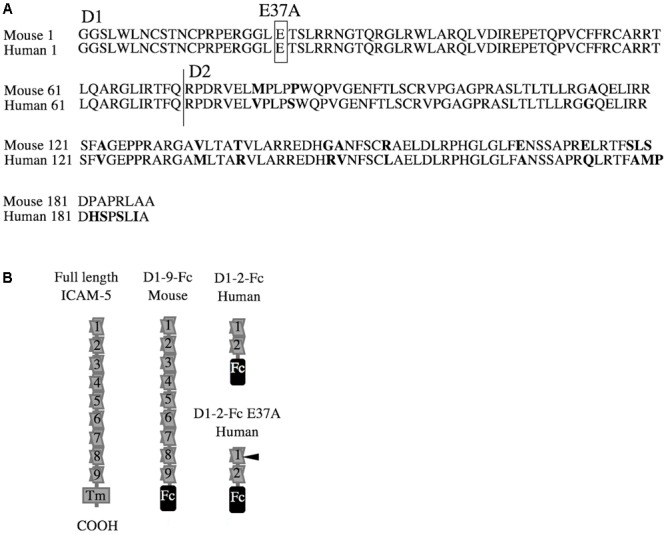
Partial sequences and purified proteins used in this study. Intercellular adhesion molecule-5 (ICAM-5) shows a high homology between species. The Ig-like domains 1–2, D1-2, that bind integrins are 90.2% identical in mice and humans. **(A)** The Ig domains are marked by D1 and D2, and the mutated glutamate is boxed and marked by E37A. An overview of the recombinant ICAM-5 proteins, all fused to the human Fc-tag, used in this study are illustrated in **B**. The E37A mutation site is indicated by a black arrow.

The human ICAM-5 D1-2-Fc E37A mutation was cloned using site-directed mutagenesis (**Figures 1A,B**; Artimo et al., 2012). The ICAM-5-Fc D1-2 and D1-2 E37A were prepared as previously described (Tian et al., 1997; Ning et al., 2013). One additional step was added to the purification protocol. The isolated proteins were run through a Pierce^TM^ High Capacity Endotoxin Removal Spin Column (Thermo Fisher Scientific). Recombinant mouse ICAM-5 D1-9-Fc chimeric protein, recombinant mouse ICAM-2-Fc chimeric protein, recombinant mouse ICAM-1 D1-5-Fc chimeric protein and recombinant mouse vascular cell adhesion molecule, VCAM-1-Fc chimeric protein were purchased from R&D Systems (Minneapolis, MN, United States). Human fibrinogen was purchased from Calbiochem (San Diego, CA, United States). Fibronectin was isolated from human plasma using gelatin agarose (Vuento and Vaheri, 1979). Recombinant purified human complement factor iC3b was purchased from Merck Millipore. Human IgG1 as a negative control was purchased from Sigma–Aldrich.

Other materials include phagocytosis beads (Fluoresbrite^TM^ carboxy YG 2.0 μm Microspheres) from Polysciences, Inc. (Warrington, PA, United States), and protein A-coated polystyrene beads (SPHERO^TM^, 4.37 μm diameter) from Spherotech Inc. (Lake Forest, IL, United States).

### Animals and Cell Culture

Wild-type (WT) and ICAM-5 knock-out (KO) C57BL/6jOla mice were used in this study. The ICAM-5 KO mice were generated as complete knock-outs by gene targeting (Nakamura et al., 2001). The animals were housed at the animal facility of the University of Helsinki with 12 h light cycle and 12 h dark cycle, food and water *ad libitum*. All the animal experiments were performed under the internal license of the University of Helsinki. Primary neuronal cultures were prepared as described (Nyman-Huttunen et al., 2006) and maintained in full Neurobasal medium (Gibco, Thermo Fisher Scientific) supplemented with 1% L-glutamine (Lonza, Basel, Switzerland) and 1x B-27 supplement (Gibco). Primary microglia cultures were prepared as described (Maciejewski-Lenoir et al., 1999). Briefly, P3-6 brains were stripped of meninges and cortical single cell suspensions were prepared. The mixed glia were cultured in poly-L-lysine (PLL, Sigma–Aldrich) pre-coated T-75 (Sigma–Aldrich) flasks until confluent, after which microglia were isolated by orbital shaking. This produced *a* > 95% pure microglia culture. BV-2 is a murine microglia cell line that was a kind gift from Heikki Rauvala, University of Helsinki. BV-2 cells were maintained in the same conditions as primary microglia in full Dulbecco’s modified eagle medium (DMEM, Gibco), 10% fetal bovine serum (FBS, Gibco), 1% penicillin-streptomycin (PS, Lonza) and 1% L-glutamine (Lonza), at +37°C and 5% CO_2_.

### Neuron-Conditioned Medium

At 14–21 days *in vitro* (DIV) cortical neuron cultures were treated for 6 h with Hank’s balanced salt solution (HBSS, Gibco) containing 1.8 mM CaCl_2_, with or without 20 μM NMDA (Sigma–Aldrich), with or without 20 μM GM6001 (Calbiochem). Prior to stimulation, the cell cultures were washed once with HBSS. The conditioned medium was filtered (0.22 μm pore size, Sartorius, Göttingen, Germany), aliquoted and frozen, unless used immediately. Normal neuron-conditioned culture medium was also used in this study. At 21 DIV, the culture medium (full Neurobasal) that had been conditioned for 3–4 days on the neurons was collected and treated as above. The neuron-conditioned medium was used to treat microglia or BV-2 cells for 1 h in the cell culture incubator.

### Fixation, Cleared Lysates and Brain Homogenates

After treatments, the cells were washed in phosphate buffered saline (PBS, Lonza) and either lysed or fixed. Cleared lysates were prepared by incubating the cells in 2% RIPA buffer (150 mM NaCl, 50 mM Tris, 10 mM MgCl_2_, 5 mM EDTA, 1% Triton X-100, 1% NP-40, pH 7.8) supplemented with fresh 1× protease inhibitor cocktail (Roche, Basel, Switzerland) and 1× phosphatase inhibitor (Roche) for 20 min on ice. Lysates were cleared to cell free extracts (CFE) by centrifugation at 16000 *g* for 20 min at +4°C. Alternatively, cells were fixed for 20 min at ambient temperature in 4% paraformaldehyde (PFA, J.T. Baker Inc., Center Valley, PA, United States) in PBS, then washed and stored in PBS at +4°C. The cortices of adult WT mice were homogenized in 2% RIPA buffer using a plastic pestle. The homogenates were cleared the same way as the lysates. The total protein concentration was measured by the Pierce BCA protein assay kit (Thermo Fisher Scientific) and 400 μg of total protein was used for IP.

### Adhesion Assays

#### RTCA iCELLigence

iCELLigence^TM^ (ACEA Biosciences, Inc., San Diego, CA, United States) is an instrument that measures electrical impedance at the bottom of a cell culture well containing microelectrodes (e-plate). The wells of the e-plate were coated with recombinant protein at 100 nM in PBS overnight at +4°C. The wells were washed with PBS and equilibrated in the cell culture incubator together with the instrument. An equilibration sweep was done followed by the addition of 20000 cells in full DMEM to each well. The experiment was started immediately with one sweep/min for the total of 6 h. The data were analyzed with the iCELLigence software and in Microsoft Excel where all individual samples were set to start from the cell index zero. The cell index is a measure of impedance recorded by the instrument. The higher the value the more spread are the cells on the bottom of the well.

#### Static Adhesion Assay

Acid-base-washed cover slips, 8-chamber slides (Thermo Fisher Scientific^TM^ Nunc^TM^ Lab-Tek^TM^ II Chamber Slide^TM^ System) or the wells of a 96-well flat-bottom microtiter plate (Greiner, Sigma–Aldrich) were coated with recombinant protein at 100 nM in PBS for 1 h at ambient temperature or overnight at +4°C. The plate was equilibrated in the cell culture incubator prior to washing with PBS and the addition of cells. BV-2 cells or primary microglia (10000 cells per well) were re-suspended in pre-warmed, full DMEM medium and allowed to interact with the substrate for 24 h in the cell culture incubator. The cells were imaged, then the culture medium was collected and cells were washed three times in PBS and fixed. ICAM-1 blocking antibody or isotype control was also added to cells cultured on ICAM-5 coated surface at 10 μg/ml.

To investigate the effect of soluble ICAM-5 on BV-2 adhesion to iC3b, flat-bottom 96-well microtiter plates were coated with iC3b (8 μg/ml in PBS) overnight at +4°C. The wells were washed three times with PBS and blocked with 1% heat-denatured (15 min at +80°C) bovine serum albumin (BSA, R&D Systems) for 1 h. Static adhesion was performed in warm HBSS buffer containing 15 mM HEPES, 5 mg/ml glucose and 1 mM sodium glutamate. The cells were pre-incubated for 10 min with 100 nM of recombinant protein, and then added to the iC3b-coated wells at the density of 120000 per well and incubated for 30 min in the cell culture incubator. Unbound cells were removed by washing twice in PBS and the adhered cells were quantified by using alkaline phosphatase assay. Briefly, *p*-nitrophenyl phosphate substrate tablets (Sigma–Aldrich) were dissolved in the buffer containing 50 mM NaCH3COO, pH 5, and 1% Triton X-100 at a concentration of 3 mg/ml, and 100 μl of substrate solution was added to each well. The cells were lysed for 45 min at +37°C. The reaction was stopped with 1 M NaOH and the optical density at 405 nm was measured (Multiskan GO Microplate Spectrophotometer, Thermo Fisher Scientific).

#### Ligand Coated Bead Adhesion

Protein A-coated beads were coupled to ICAM-5-Fc D1-9, D1-2-Fc, D1-2 E37A-Fc or ICAM-1-Fc. One microgram of protein per 10 μl bead slurry was allowed to couple for 1 h at +4°C. Unbound proteins were washed away once with PBS. Primary microglia or BV-2 cells cultured in 24-well plates were washed once in PBS, and the 10 μl of coated bead slurry in 300 μl PBS was added. Cells were allowed to bind beads for 1 h in the cell culture incubator. The unbound beads were then rigorously washed away with PBS and the cells were fixed and stained with phalloidin, and anti- β1- and β2-integrin antibodies.

### Pull-Down Assay

BV-2 cells were cultured until approximately 80% confluent in 10 cm culture plates. Lysates were prepared and the corresponding amount of one plate was used per sample. One microgram recombinant protein was coupled to 20 μl protein G slurry (GE Healthcare, Little Chalfont, United Kingdom) and washed in PBS. The CFEs that had been combined for pre-clearing were divided over the tubes containing the coupled sepharose. After a 2 h incubation at +4°C with end-over-end rotation, the sepharose was washed twice in 2% RIPA buffer and then once in PBS. The protein complexes were eluted by boiling for 5 min in Laemmli sample buffer. The samples were then subjected to WB analysis.

### Immunoprecipitation

BV-2 cells treated for 1 h with neuron-conditioned medium were lysed and the pooled CFE was pre-cleared. The corresponding volume of CFE from one 10 cm culture plate (1 ml) was incubated for 2 h at +4°C with 1 μg of one of the capture antibodies. Twenty microliter of protein G slurry was added to each sample that were then incubated for 1 h at +4°C. Alternatively, 400 μg total protein of adult WT cortical homogenates were used for IP using 179D as capture antibody. The sepharose samples were washed twice in 2% RIPA buffer and once in PBS. The antibody complexes were then eluted by boiling for 5 min in Laemmli sample buffer. The samples were further analyzed by WB.

### Western Blots

Samples were separated by SDS–PAGE in a NuPAGE^TM^ 4–12% Bis-Tris Protein Gel, 1.0 mm, 10-well (Thermo Fisher Scientific) and transferred to nitrocellulose (GE Healthcare) or polyvinylidene difluoride (PVDF, Thermo Fisher Scientific) membranes. Precision Plus Protein^TM^ Pre-stained All Blue standard (Bio-Rad, Hercules, CA, United States) was used. The membranes were then blocked in 5% skimmed milk, followed by primary antibody in 3% skimmed milk for 2 h at ambient temperature or overnight at +4°C, then after washing, incubated for 1 h at ambient temperature with secondary HRP-conjugated antibody. After the final washing steps, the membranes were incubated in Clarity ECL substrate (Bio-Rad) and used to expose x-ray films (Fuji film, Tokyo, Japan). The band intensities were analyzed using Fiji (NIH).

### Immunofluorescence Staining

Fixed cells were permeabilized with PBS containing 0.1% saponin (Sigma–Aldrich) for 5 min. They were then blocked with PBS containing 0.05% saponin and 5% BSA for 2 h at ambient temperature or overnight at +4°C. Antibodies were diluted in PBS with 3% BSA and 0.05% saponin. Primary antibody incubation was for 2 h at ambient temperature or overnight at +4°C. Secondary antibody incubation was carried out for 1 h at ambient temperature. Washing steps following antibody incubations were three times for 10 min with PBS containing 0.05% saponin. Cover slips were finally mounted with ProLong gold or diamond antifade reagent (Thermo Fisher Scientific).

### Phagocytosis Assay

The phagocytic capacity of microglia was investigated in cultured microglia. Isolated microglia were cultured for 3–5 days on PLL-coated glass cover slips or non-coated cell culture plastic. Phagocytosis beads were mixed 1:100 in full DMEM containing 100 nM of protein. The cells were incubated for 1 h with the beads and the protein in the cell culture incubator. The cultures were thoroughly washed with PBS prior to fixation.

### ELISA Assay

The mouse TNF-α, CCL5, IL-1β and IL-10 DuoSet ELISA were purchased from R&D Systems. Culture medium from microglia treated for 24 h with DMEM supplemented with 5% FBS, 1% L-glutamine, 10 ng/ml LPS (Sigma–Aldrich) and either ICAM-5 D1-9 or D1-2, ICAM-1, IgG or only PBS, was collected and centrifuged to remove cellular debris and stored at -80°C until further use. Hundred microliter of the medium was analyzed for cytokine production in duplicates according to the manufacturer’s instructions. The optical density at 450 nm was measured.

### Imaging and Data Analysis

Cells were imaged with the confocal microscope Leica TCS SP5 fitted with the HCX APO 63×/1.30 Corr (glycerol) CS 21objective or Evos inverted microscope (Thermo Fisher Scientific). Where applicable, experiments were run in at least duplicates and repeated at least three times with similar results. Micrographs were collected at least 10 frames per sample. The data set was blinded and analyzed. The data are represented as normalized between experiments or single experiments where repetitions show similar results.

Images were analyzed using Fiji (NIH). For co-localization studies, confocal images were automatically analyzed and individual cells were manually selected as region of interest. The data were analyzed using JASP software. The data are shown as mean values with standard deviation as error bars. Statistical significance was investigated by non-paired Student’s *t*-test or one-way ANOVA, followed by Bonferroni’s *post hoc* test (^∗^*P* < 0.05, ^∗∗^*P* < 0.01, and ^∗∗∗^*P* < 0.001).

## Results

### Endogenous ICAM-5 from Neurons Is Bound by Microglia

**FIGURE 2:**
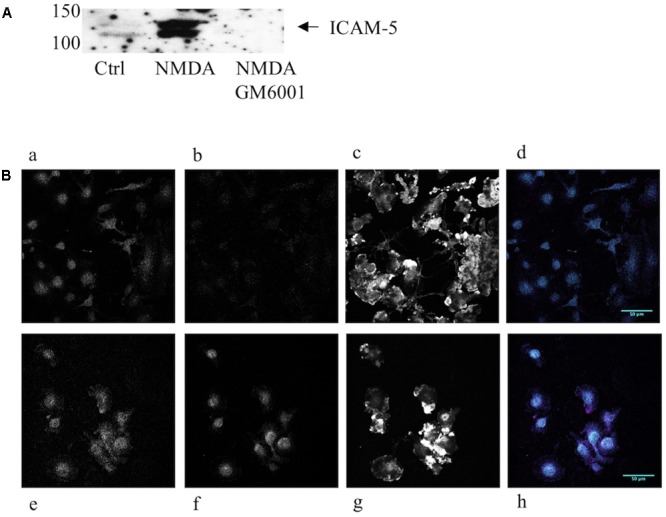
Endogenous neuronal ICAM-5 is bound by microglia *in vitro*. **(A)** ICAM-5 is released into the culture medium as is illustrated by the WB of WT neuron-conditioned culture medium where cells were treated with HBSS with or without NMDA, with or without GM6001. **(B)** Microglia treated for 1 h with WT neuron-conditioned HBSS with **(Be–h)** or without NMDA **(Ba–d)** were IF stained for integrin β2 (magenta, **Ba,e**), ICAM-5 (cyan, **Bb,f**) and F-actin **(Bc,g)**. **Bd**,**h** are merges of β2 and ICAM-5, scale bar = 50 μm. The strong increase in ICAM-5 staining in **Bf** indicates that ICAM-5 has been bound by microglia.

The intercellular adhesion molecule-5 is solubilized by strong synaptic activity. To investigate the biological activity of the soluble ICAM-5, we studied the effect on microglia. Microglia are involved in pruning and the most active time for pruning happens to coincide with a rapid increase in ICAM-5 expression in the mammalian brain (Yoshihara and Mori, 1994). First, we wanted to verify that endogenous soluble ICAM-5 released from neurons could be bound by microglia. For this purpose, we prepared neuron-conditioned medium from WT and ICAM-5 KO cortical neuronal cultures. As illustrated in **Figure 2A**, cultured neurons constitutively shed ICAM-5 into the culture medium. This effect was dramatically enhanced by NMDA treatment of the cells and the general MMP inhibitor, GM6001, completely abrogated the release of ICAM-5, as has been shown previously (Tian et al., 2007). Microglial cells that had been treated with WT neuron-conditioned HBSS supplemented with NMDA were found to be positive for ICAM-5, indicating that the cells were capable of binding soluble fragments of ICAM-5 (**Figure 2B**). Microglia treated with ICAM-5 KO conditioned HBSS supplemented with NMDA was negative for ICAM-5. The experiments mentioned above provided evidence that ICAM-5 associated with β2 integrins on microglia. The co-localization between ICAM-5 and β2 was more than moderate (Pearson’s correlation coefficient 0.624, standard deviation 0.058, calculated from cells represented in **Figure 2Bh**).

**FIGURE 3:**
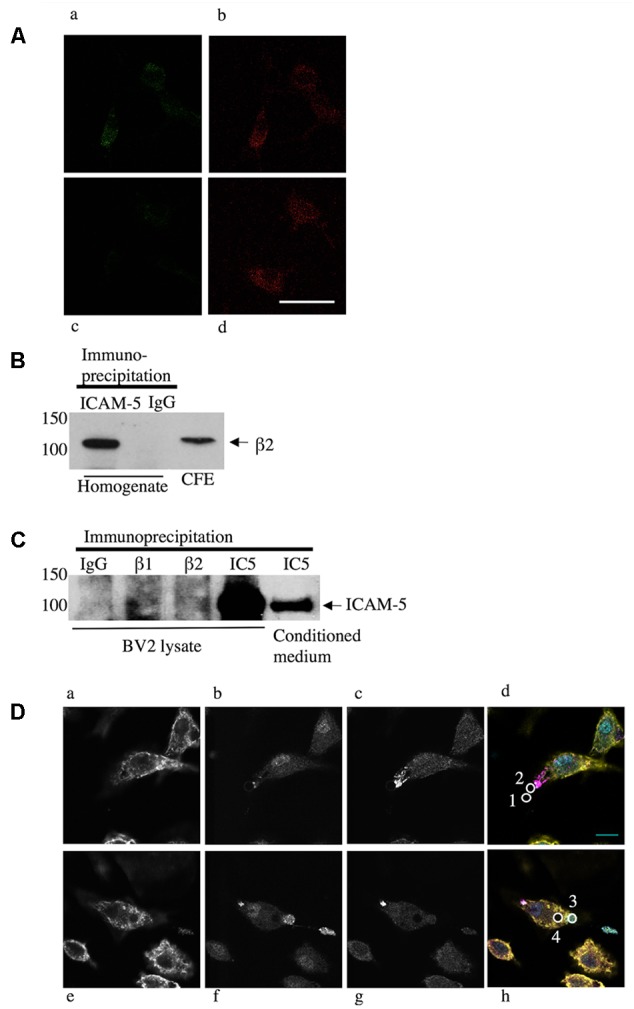
Endogenous neural ICAM-5 is bound by microglia *in vitro* and it associates with integrins *in vivo*. **(A)** Primary microglia from WT mice were treated with WT **(Aa,b)** or ICAM-5 KO **(Ac,d)** neuron-conditioned culture medium and stained for ICAM-5 **(Aa,c)** and β2 **(Ab,d)**. Scale bar = 10 μm. ICAM-5 and β2 also associate in cortical homogenates of adult C57Bl/6j mice when ICAM-5 is immunoprecipitated and the complex is blotted for β2 in **B**. BV-2 cells as well bind ICAM-5, demonstrated by an IP of BV-2 cells treated with WT neuron-conditioned medium in **C**. The IP antibodies are presented on the top (IC5 = ICAM5) and the ICAM-5 antibody 246H is used as detection antibody. Recombinant ICAM-5 D1-9 coupled to beads was bound by microglia. **(D)** Microglia were allowed to interact with ICAM-5 D1-9 coated beads and then stained for F-actin (yellow, **Da**,**e**), β1 integrins (cyan, **Db**,**f**), β2 integrins (magenta, **Dc**,**g**). In **Dd**,**h**, the figures **a–c** or **e–g** are merged and the beads are illustrated by white circles that are numbered 1–4. Bead 1 represents a bead not bound by a cell, bead 2 is in the first stage of contact, recruiting β2 integrins, bead 3 is captured by the cell, employing β1 integrins, and bead 4 is completely internalized. Scale bar = 10 μm.

Soluble ICAM-5 bound to microglia and co-localized to the β2 integrin chain (**Figure 3A**). To further study this, we immunoprecipitated ICAM-5 from the adult mouse brain and detected a significant amount of β2 integrin in the immune-complex (**Figure 3B**). ICAM-5 also associates with β1 *in vivo* (Ning et al., 2013). Neither β1 nor β2 integrins are exclusively expressed by microglia in the brain, even though microglia are the main cell type expressing β2. To investigate the role of β1 and β2 integrins in ICAM-5 binding in microglia, BV-2 cells were treated with WT neuron-conditioned culture medium and subjected to IP. A weak but significant association of ICAM-5 with both β1 and β2 integrins could be seen (**Figure 3C**). To avoid variance in protein expression levels, the CFE was pooled for pre-clearing before incubation with the IP antibodies. Furthermore, endogenous soluble ICAM-5 from neurons was strongly bound by BV-2 cells, as seen by immunoprecipitating ICAM-5 (**Figure 3C**). Lysates of unstimulated BV-2 cells did not express ICAM-5 (data not shown) and microglia treated with ICAM-5 KO conditioned medium were negative for ICAM-5, which is in line with previous findings that ICAM-5 is neuron-specific (Mitsui et al., 2005). The morphology of microglia treated with WT or ICAM-5 KO neuron-conditioned HBSS with NMDA for 2 h was also quantified but no significant differences were found.

To further characterize the binding of ICAM-5 to microglia, recombinant proteins were used. We used ICAM-5 coated beads and allowed microglia to interact with these for 1 h prior to fixation and IF staining. **Figures 3Da,e** show actin staining (yellow), **Figures 3Db,f** show integrin β1 staining (cyan), and **Figures 3Dc,g** show integrin β2 staining (magenta). To quantify the involvement of integrins proved challenging, since integrins are very dynamic and their recruitment around the beads seems to be transient. During the 1 h experiment, most of the bound beads were internalized completely. Only at the initial contact site between the beads and the cells corresponding to a small fraction of the beads, integrins were observed to form clusters (**Figure 3Dd**, bead number 2). Beads that were already completely internalized seemed to lose the integrin clustering (**Figure 3Dh**, bead number 4). Beads not bound to the cells were negative for β1 and β2 (**Figure 3Dd**, bead number 1) and completely internalized beads can be seen as lack of staining, especially clear by the actin staining (**Figure 3De**). Thus, integrins seem to be involved in the capturing of the bead, with β2 binding first, followed by β1.

**FIGURE 4:**
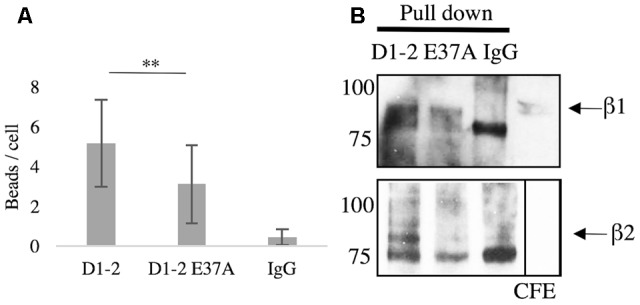
Glutamate-37 is important in ICAM-5 binding to β2 integrins. **(A)** Microglia were allowed to interact with ICAM-5 D1-2 WT or D1-2 E37A coated beads for 1 h and were then washed. Beads coated with D1-2 E37A were significantly less bound as compared to the D1-2 WT (^∗∗^*P* < 0.01). The D1-2 E37A protein was found to bind β1 but not β2 integrins in the pull-down assay, shown in **B**. Separate pull-down eluates were blotted for β1 in the top panel and β2 in the lower panel. The proteins used as baits are indicated on top of the panels.

Since glutamate-37 in ICAM-5 has been shown by crystallography to be important for the binding to LFA-1 (Zhang et al., 2008), we wanted to explore this experimentally. Beads were coated with D1-2 WT or D1-2 E37A mutant. ICAM-5 is known to bind both β1 and β2 integrins through the first two domains (Tian et al., 2000a; Ning et al., 2013). Comparing D1-2 WT and E37A, the mutation significantly reduced the binding capacity (^∗∗^*P* < 0.01, **Figure 4A**). As illustrated by the pull-down assay in **Figure 4B**, we could further see that both WT and E37A D1-2 were able to bind β1 integrins. However, only the WT could pull down β2 from BV-2 lysates, but the β2 integrin is expressed at low levels.

### ICAM-5 Induces Clustering and Weak Adhesion of Microglia

**FIGURE 5:**
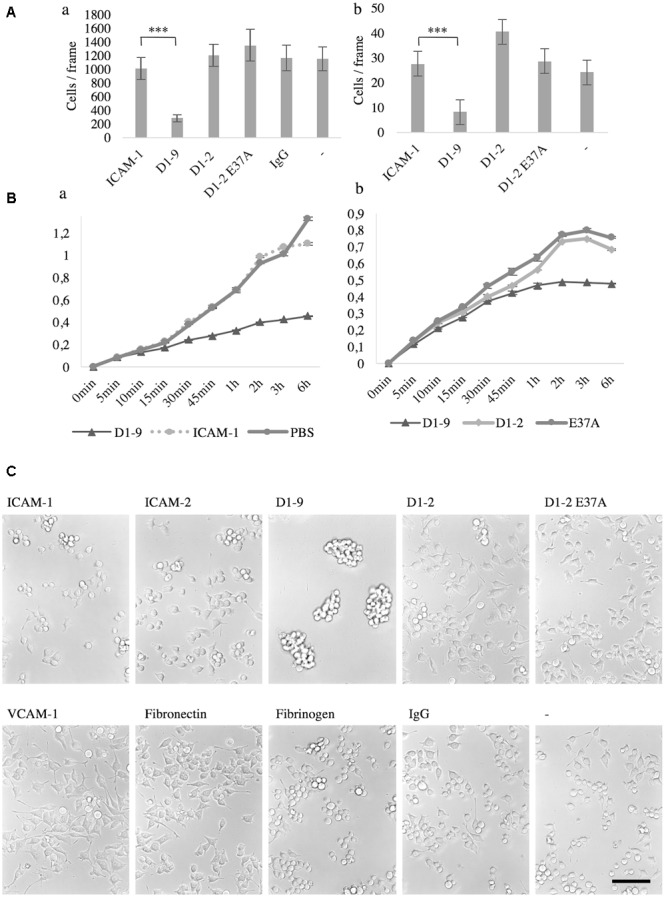
The intercellular adhesion molecule-5 D1-9 coated surface is anti-adhesive and induced clustering of unstimulated microglia and BV-2 cells. Cells that remain after washing when cultured for 24 h on ligand coated surface. **(A)** The number of cells per frame remaining after washing was quantified after staining with DAPI. **Aa** represents BV-2 cells, while **Ab** is primary microglia (^∗∗∗^*P* < 0.001). The spreading of microglia on a ligand coated surface was also measured by iCELLigence **(Ba,b)**. The cell index was generated by the instrument as a value of impedance. The higher the number, the more spread the cells are. In **C**, BV-2 cells were cultured for 24 h on various pre-coated surfaces as shown in the figure. The cells were imaged without washing and it is clear that cluster formation was specifically induced by the ICAM-5 D1-9 coating. Scale bar = 100 μm.

Now knowing that microglia bind ICAM-5, we were curious to study the effect of this binding further. First, we wanted to see if there were any gross differences in microglia morphology and adhesiveness to ICAM-5 D1-2 and D1-9 as compared to ICAM-1 in unstimulated cells. Using the static adhesion assay, BV-2 cells and microglia were found to weakly adhere to ICAM-5 D1-9-coated surfaces as compared to all other ligands tested. After washing, most of the cells cultured for 24 h on ICAM-5 D1-9 were removed (**Figure 5Aa**). The same effect was observed with primary microglia (**Figure 5Ab**). The RTCA iCELLigence instrument was used as an unbiased method to measure cell spreading. Again, ICAM-5 D1-9 induced much less spreading of microglia as compared to ICAM-1 (**Figure 5Ba**) or ICAM-5 D1-2 WT or D1-2 E37A (**Figure 5Bb**). The effect was rapid and was seen already 15 min after adding the cells to the coated wells. The most prominent effect was observed after 3 h. To explain the interesting effect of ICAM-5 D1-9, the experiment was repeated in cell culture plates and cells were imaged after 24 h without washing (**Figure 5C**). At this point, it was clear that on ICAM-5 D1-9 coated surface, BV-2 cells formed clusters. These clusters did not adhere to the substrate and are easily washed away. This assay was used to screen for similar effects of other adhesion molecules (ICAM-1, ICAM-2, VCAM-1, fibronectin and fibrinogen), but strong clustering was seen only for ICAM-5 D1-9. BV-2 spread poorly on fibrinogen, and the cells appeared round and importantly they did not form cell clusters. ICAM-1 function blocking antibodies could not abolish the formation of cell clusters, indicating that it is not involved in this process (data not shown).

### ICAM-5 Downregulates Phagocytosis in Unchallenged Microglia

**FIGURE 6:**
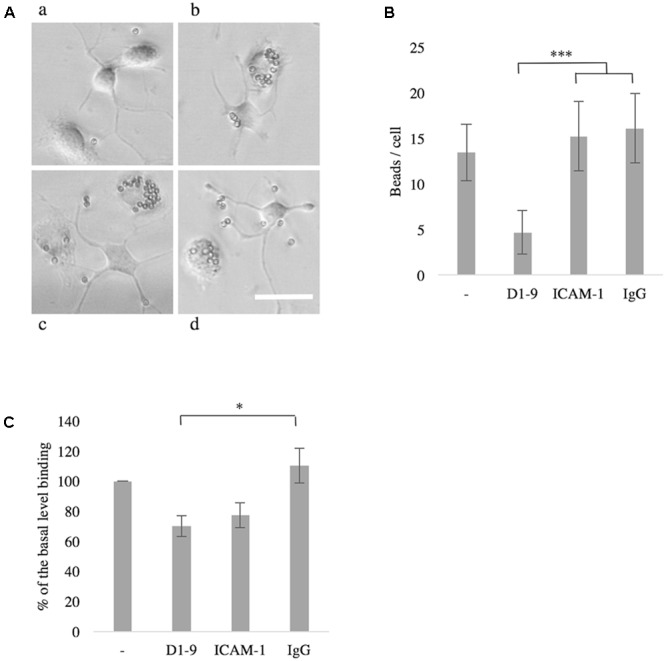
The intercellular adhesion molecule-5 inhibits phagocytosis by unchallenged microglia or BV-2 cells. Microglia treated with soluble ICAM-5 D1-9 were significantly less efficient in phagocytosing latex beads (**Aa**, quantified in **B** as number of beads per cell, ^∗∗∗^*P* < 0.001) as compared to cells treated with human IgG **(Ab)**, ICAM-1 **(Ac)** or PBS alone **(Ad)**. Scale bar = 25 μm. Soluble ICAM-5 also inhibited BV-2 cell adhesion to iC3b coated surface **(C)** (^∗^*P* < 0.05). All microglia were seeded on iC3b coated surface supplemented with soluble proteins as indicated. After 30 min the wells were washed and the remaining cells were quantified by alkaline phosphatase assay. Cells not supplemented with any protein (first bar in the graph) were normalized to 100, representing the basal binding.

Phagocytosis is an essential function of microglia in both health and disease. We saw a down-regulation of phagocytosis of small latex beads when unstimulated microglia were treated with soluble ICAM-5, as compared to soluble ICAM-1, human IgG or non-treated cells (*P* < 0.001, **Figures 6A,B**). Soluble ICAM-5 D1-9 could also inhibit BV-2 cell binding to coated iC3b as compared to soluble IgG (*P* < 0.05, **Figure 6C**). Basal binding of BV-2 cells to iC3b in the absence of any additional soluble proteins was normalized to 100 and the presence of soluble ICAM-5 reduced this binding by approximately 30% and by 40% as compared to soluble IgG.

### ICAM-5 Reduces TNF-α and IL-1β but Promotes IL-10 Secretion from LPS-Stimulated Microglia

**FIGURE 7:**
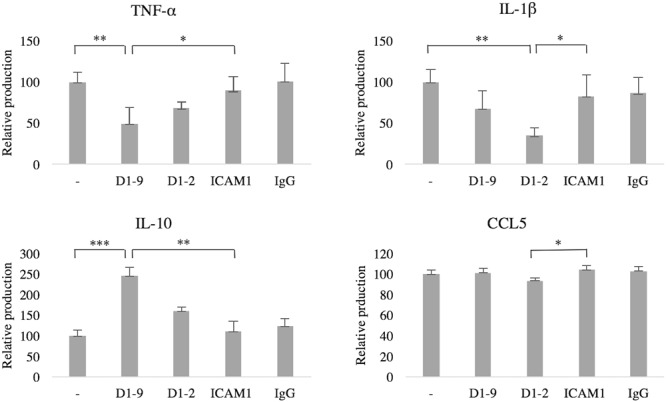
Soluble ICAM-5 reduces TNF-α and IL-1β but induces IL-10 secretion under LPS challenge. ICAM-5 had a minor effect on CCL5 secretion. All microglia were treated for 24 h with LPS and with soluble proteins as indicated. The cytokine production of cells in LPS only (first bar in each graph) was normalized to 100, representing the induced production of each cytokine. The culture medium was analyzed for TNF-α, IL-1β, CCL5 and IL-10 using ELISA. TNF-α secretion was significantly reduced when ICAM-5 D1-9 was present, as compared to controls [ICAM-1 (^∗^*P* < 0.05) or LPS only (^∗∗^*P* < 0.01)]. ICAM-5 D1-2 could also reduce IL-1β secretion, as compared to ICAM-1 (^∗^*P* < 0.05) or LPS only (^∗∗^*P* < 0.01). The anti-inflammatory cytokine IL-10 was on the other hand upregulated by ICAM-5 D1-9, as compared to ICAM-1 (^∗∗^*P* < 0.01) or LPS only (^∗∗∗^*P* < 0.001).

Finally, we wanted to investigate the immunological implications of ICAM-5 on microglia. Cytokine secretion into the culture medium was measured by ELISA. Without LPS-stimulation, cytokine production was negligible (data not shown), therefore the cells were treated with LPS and the effect of ICAMs was studied. The secretion of TNF-α was reduced by soluble ICAM-5 D1-9 (*P* = 0.03), however, ICAM-5 D1-2 showed no significant effect (*P* = 0.79, **Figure 7**). ICAM-5 D1-2 significantly reduced the secretion of IL-1β into the culture medium (*P* = 0.03), whereas ICAM-1 had no effect. Interestingly, ICAM-5 D1-9 only slightly reduced the secretion. However, the secretion of the anti-inflammatory cytokine IL-10 was strongly increased when cells were treated with soluble ICAM-5 D1-9. ICAM-5 D1-2 only marginally reduced the secretion of CCL5.

## Discussion

We have now shown that microglia bind ICAM-5 *in vitro*. This interaction is important to characterize since it is confined to the brain and links neuronal functions to the immune system.

In unchallenged microglia, soluble ICAM-5 impaired phagocytosis of uncoated modified latex beads. Additionally, adhesion to the complement factor iC3b was inhibited by soluble ICAM-5. iC3b is an important mediator of phagocytosis and C3 is involved in tagging synapses for pruning (Stevens et al., 2007). It is possible that the soluble ICAM-5 released from active synapses can reduce phagocytosis locally and protect active synapses from being pruned. Proteins of the FBS, such as vitronectin could play a role here (Furutani et al., 2012).

Comparing the effects of ICAM-1 versus ICAM-5 is interesting. In the brain, ICAM-1 localizes mainly to glia, endothelial and epithelial cells, while ICAM-5 is expressed by telencephalic neurons (Tian et al., 2008). The expression of ICAM-1 in the brain is regulated by cytokines and ICAM-1 is generally up-regulated under inflammatory conditions. Leukocyte binding to ICAM-1 usually requires activation of the cells. Both ICAM-1 and ICAM-5 can be solubilized and have as such quite opposite effect on T-cells and macrophages. While ICAM-1 is proinflammatory in macrophages (Schmal et al., 1998), ICAM-5 was shown to be rather immune-suppressive in T-cells (Tian et al., 2008). The M1/M2 paradigm has been adopted from the peripheral immune system to categorize microglia polarization, but it has proven to be insufficient in explaining microglia inflammatory states. Many of the molecules implicated in either M1 or M2 have different effects on neuronal plasticity in the healthy brain (Donzis and Tronson, 2014). For example, TNF-α is in a concentration dependent manner important for learning and memory. We activated microglia with LPS, which is a classical molecule rendering microglia the M1 phenotype. TNF-α and IL-1β secretion from these cells was decreased when the cells were co-stimulated with soluble ICAM-5, while the anti-inflammatory IL-10 was upregulated. ICAM-1 is known to induce TNF-α secretion in macrophages (Schmal et al., 1998). In this case, however, all samples were LPS-stimulated, which could mask the proinflammatory effect of ICAM-1. Together, these data strongly suggest that ICAM-5 suppresses the M1 response or even tilt the phenotype toward M2. IL-10 has previously been shown to reduce TNF-α secretion, which could explain the observed reduction of TNF-α (Bolger et al., 2002). The anti-inflammatory effect of ICAM-5 on microglia indicates that it has an important neuroprotective role.

The intercellular adhesion molecule-5 is solubilized under several pathological conditions, such as encephalitis, and soluble ICAM-5 is found in the serum of patients (Lindsberg et al., 2002). ICAM-5 has a proven potential of modulating T-cell responses and could function both locally in the brain and systematically through the blood circulation. This poses an interesting therapeutic potential for ICAM-5 that should be explored in the future. ICAM-5 has been found to be internalized from the membrane through endocytosis (Raemaekers et al., 2012). The presence of ICAM-5 in extracellular vesicles has not been studied, but it is more likely that ICAM-5 is solubilized in free form at least in the NMDA-MMP-related mechanism.

BV-2 cells formed large cell clusters when seeded on ICAM-5-coated surfaces, as compared to all other proteins tested. Neither primary microglia nor BV-2 cells were able to form firm adhesion and spread on D1-9 coated surfaces. ICAM-5 seems to be anti-adhesive; however, we cannot exclude that the ICAM-5 ectodomain is not repulsive *per se*, but instead it induces a signaling cascade that promotes surface expression or activation of adhesive molecules on microglia. It seems possible that ICAM-5 D3-9 could contain binding sites to a yet unknown receptor that induces this signaling. It was generally difficult to get direct and exclusive evidence of either β1 or β2 integrins binding to ICAM-5 *in vitro*, further strengthening the possibility of other receptors involved in the binding of ICAM-5 to microglia. Soluble ICAM-5 was efficiently internalized by microglia *in vitro* and endogenous ICAM-5 was not detected at least in BV-2 cells. Previous studies have seen ICAM-5 immunoreactivity *in vivo* in microglia (Kelly et al., 2014). However, it was not defined whether this protein originated from endogenous expression or if it was neuron derived and internalized through scavenging.

*In vivo* studies have shown that microglia tends to make direct contact with the spine heads, rather than the dendritic shaft (Wake et al., 2009). ICAM-5 is abundantly expressed on the dendritic shafts and filopodia, while it is expelled from the mature spine heads (Matsuno et al., 2006). This repulsive effect of the dendritic shafts could be conveyed by full-length ICAM-5 that is abundant in the membrane. ICAM-5 may actually function similarly to the developmental endothelial locus-1 (Del-1). Del-1 is a ligand for LFA-1 and strongly expressed in the brain. By binding to this receptor, it reduces the LFA-1 binding to ICAM-1 and thereby limits adhesion of neutrophils to endothelial cells (Choi et al., 2008). Del-1 has recently been found to be anti-inflammatory in multiple sclerosis (Choi et al., 2015), further strengthening the functional similarities between these molecules. Our results are, however, contradictory to the previous findings that microglia spread by addition of ICAM-5 (Mizuno et al., 1999). We noted that microglia are very sensitive to the presence of endotoxin in adhesion studies and using the endotoxin removal column was essential. Furthermore, the effect of ICAM-5 seems to be concentration dependent and coated versus soluble administration can have vastly different effects, demonstrating the versatility of this molecule.

In the current study, ICAM-5-coated particles were bound by microglia. Especially the beads coated with only the two most rostral Ig-domains of ICAM-5, D1-2, were prominently bound by microglia. This might be due to a different conformation of ICAM-5. The avidity of the molecule can potentially be modulated by masking of binding sites or by homophilic binding between molecules. When ICAM-5 is coupled to the beads, the homophilic binding of D1-9 is presumably reduced on the surface of the bead and more D1-2 sites are exposed. Coating with only D1-2 further elevates the amount of binding sites. D1-2 is also positively charged (Zhang et al., 2008; Recacha et al., 2014), a trait that is known to facilitate internalization of particles in phagocytic cells. When ICAM-5 D1-9 is coated on a larger surface, it might be present in polymers due to homophilic binding, which can have different binding and signaling characteristics. We could additionally show, that the functional binding of ICAM-5 to microglia was impaired when glutamate-37 was mutated to alanine. The pull-down assay revealed that only β2 binding is impaired, β1 less so. The IF staining of β1 and β2 integrins in cells binding ICAM-5 coated beads provided clues that these integrins are recruited at different stages of the capturing and internalization of cargo. Since the bead binding was impaired in the case of the mutant, it seems possible that the initial recruitment of β2 integrins in the capturing of the particle is not essential in this type of phagocytosis and additional receptors might play a role here.

## Conclusion

The intercellular adhesion molecule-5 is a unique adhesion molecule in its binding capacity, flexibility and various potential signaling abilities. As compared to the other members of the ICAM family, ICAM-5 is the only one that is predominantly expressed in the brain. It is possible, that ICAM-5 has different effects on microglia, depending on whether the brain is under development or immune challenge. Soluble and membrane-bound forms of ICAM-5 seem to have different functions. ICAM-5 is a versatile molecule and serves neurons differently during development, promoting neurite sprouting, while in mature neurons, it is a negative regulator of synaptic maturation. In the aged brain, it might play a role in the pathophysiology of Alzheimer’s disease (Annaert et al., 2001). This raises again the interest to the role of microglia and the interaction with ICAM-5.

## Ethics Statement

This study was carried out in accordance with the recommendations of the National Animal Experiment Board in Finland. The protocol was approved by the University of Helsinki.

## Author Contributions

SP planned, executed, and analyzed experiments and drafted the manuscript and the figures, contributed to the concept and interpretation of the work. TR planned, executed, and analyzed experiments and contributed to the concept and interpretation of the work. LN contributed to the concept and the revision of the work. CG contributed to the concept of the work, the interpretation of the data and the writing.

## Conflict of Interest Statement

The authors declare that the research was conducted in the absence of any commercial or financial relationships that could be construed as a potential conflict of interest.
